# Philadelphia Hospital, Service of Meredith Clymer, M. D. Case of Soft Cancer of the Ovary, with a Multilocular Cystic Dropsy

**Published:** 1843-07-08

**Authors:** W. C. Benedict

**Affiliations:** Resident Physician


### PHILADELPHIA HOSPITAL.

Service of Meredith Clymer, M. D.

(Reported by W. C. Benedict, Resident Physician.)

### *Case of Soft Cancer of the Ovary, with Multilocular Cystic Dropsy.*

Caroline R., æt. 22, a German by birth, of good habits, has had one child, and always enjoyed good health, until about the 12th of January, 1843, when, while carrying a heavy weight, she felt a sudden pain in lower part of abdomen, which was followed by a discharge from vagina. The pain gradually increased from that time, but was not severe enough to confine her to bed, until about the first of March.

Entered medical ward, March 7th. General appearance of patient—cachetic, countenance sharp and expressive of anxiety; skin a little warmer than natural; pulse 110, soft, moderately full, and rather quick; tongue has a whitish coat in the middle, clean at the edges; respiration high, quick, and entirely thoracic; chest healthy, no pain, and no cough.

Abdomen much enlarged at the lower part, tense, and shining, dull on percussion, quite tender on pressure, especially above the left iliac region. On auscultation no abnormal sound was discovered. A large tumour could be felt, on passing the hand over the abdomen, filling up the whole of the cavity of the pelvis and lower part of abdomen.

On examining per vaginam, the walls were found healthy; the os, and cervix uteri, rather to the right side behind the symphysis pubis, and in immediate contact with the posterior parieties of the urethra, and in a healthy condition; posterior to the neck of the uterus a large round tumour was found, projecting partly into the vagina, of firm, uniform consistence, and quite elastic.

Patient is obliged to remain in a semi-recumbent posture, on account of the pain, and difficulty of breathing while lying. Her bowels are habitually costive; has no difficulty in passing her urine; appetite very good. Death supervened May 26th.

*Autopsy 24 hours after death.*

On inspection of the body, the abdomen was found as large, as at full term of utero-gestation. The wrists, hands, and both of the lower extremities, greatly œdematous, and pitting on pressure.

The walls of the abdomen were adherent to within three inches of the ensiform cartilage, to a firm body within, being connected by dense layers of lymph, which could be separated only with difficulty. The tumour extended from the bottom of the pelvis to the sternum, filling up the whole of the cavity, and connected to the apex of the liver by a firm adhesion; the stomach and intestines laying posteriorly, and much compressed; the ascending and descending portions of the colon with the peritoneal covering, were adherent to the tumour posteriorly, and of a dark slate colour; the arch remained free.

Numerous globular cysts were found in the liver, at or near the surface, of various sizes, consisting of soft, whitish grey matter. The left kidney was found three inches above its natural situation, and very close to the spinal column; the ureter natural; right kidney in situ, the ureter distended to an inch in diameter, and filled with urine; substance of kidneys harder than natural, otherwise healthy.

Uterus of natural size, very hard, almost cartilaginous, and adherent by its posterior surface; right ovary distended greatly with fluid, adherent to the mass, and of three times its natural size, the fimbriated extremity of the fallopian tube, studded with small tumours, similar to those found in the liver. The left ovary and fallopian tube were entirely obliterated, being merged in the tumour.

The tumour was made up of numerous cysts, of various sizes, containing dark coloured, turbid serum; the walls of the cysts consisting of encephaloid matter, presenting a whitish grey appearance, soft, and easily broken.

The quantity of fluid contained in the tumour was estimated to be about two gallons, and the tumour, exclusive of the fluid, weighed fourteen and a half pounds; viscera of the chest were healthy. Brain was not examined.

*Remarks.*—The patient was examined by Dr. Dunglison, soon after her admission, and a diagnosis of multilocular cystic dropsy of the left ovary was made, and a palliative plan recommended by him, one calculated to keep the patient's system as near the standard of health as possible. For this purpose gave mild laxatives and mucilaginous enemata *pro re nata*, to prevent constipation; the solution of morphia, at first f℥j. morning and evening; afterwards as often as indicated, and anodyne applications to abdomen, to relieve the pain; also, wine was given to support her strength.

Although numerous examinations were made during the disease to discover any fluctuation, it was not clearly made out until about three weeks before death when it was found in limited spaces, chiefly in left iliac and hypogastric regions, and apparently deep seated.

The malignant nature of the tumour was not suspected until revealed by the post mortem examination, being at no time indicated by that peculiar straw-coloured tint of the skin, so characteristic of the cancerous diathesis.

